# The Application of Clinical Nursing Pathway in the Classification Diagnosis and Treatment of Patients with Emergency Dyspnea

**Published:** 2017-04

**Authors:** Xiaowei FENG, Meihuan XIANG, Xiuna HUANG, Xiaomei FENG, Xiaoling FENG

**Affiliations:** 1. Dept. of Emergency, Sun Yat-sen Memorial Hospital, Sun Yat-sen University, Guangdong, China; 2. Dept. of Integrated, Sun Yat-sen Memorial Hospital, Sun Yat-sen University, Guangdong, China; 3. Outpatient Operating Room, Traditional Chinese Medicine Hospital of Guangdong Province, Guangdong, China; 4. Dept. of Nursing, Sun Yat-sen Memorial Hospital, Sun Yat-sen University, Guangdong, China

## Dear Editor-in-Chief

Majority of patients treated in the emergency department are cases of dyspnea (1). Medical resources in the emergency department are usually very limited. Based on emergency nursing experience of treating dyspnea, the treatment process can be optimized using evidence-based medical science to establish a scientific and normalized clinical nursing pathway that can help guide hierarchical partition nursing work (2). The clinical nursing pathway applied in Sunyat-sen Memorial Hospital has achieved a good clinical nursing effect in the hierarchical diagnosis for emergency patients with dyspnea.

The purpose of the present study was to investigate the value of the clinical nursing pathway in classification diagnosis and treatment of patients with emergency dyspnea.

A total of 124 cases of emergency dyspnea treated in our Emergency Department were consecutively selected. Patients were randomly and equally divided into the control group (n=62; general nursing mode) and observational group (n=62; clinical nursing pathway).

Our findings showed that the control group was classified into different levels, with eight cases in level I, 20 cases in level II, nine cases in level III, and 25 cases in level IV. Fifty cases (80.65%) were cured successfully. Five cases of level I, six cases of level II, and one case of level III failed to be cured. In the observational group, there were nine cases of level I, 21 cases of level II, eight cases of level III, and 24 cases of level IV. Fifty-eight cases (93.55%) were cured successfully. Two cases of level I and two cases of level II failed to be cured; no differences in medical grading were found between the two groups (Z= −0.571, *P*=0.568). The success rate in the observational group was significantly higher than in the control group (χ^2^=4.593, *P*=0.032). Taken above, significant lower nursing error rate and shorter emergency treatment residence time were found in the observational group, as shown in Table 1 (*P*<0.05).

**Table 1:** Comparison of nursing error rate and emergency treatment residence time

**Group**	**Cases**	**Nursing error rate**	**Emergency treatment residence time (min)**
**Control Group**	62	2(3.23)	41.2±10.3
**Observational Group**	62	8(12.90)	25.9±8.6
***t*/χ**^**2**^		3.916	5.629
***P***		0.048	0.007

Regarding nursing satisfaction for two nursing modes, as shown in Table 2, the total satisfaction score in the observational group was higher than in the control group (*P*<0.05). No significant differences were found in the individual comparisons between the two groups (*P*>0.05).

**Table 2:** Comparison of nursing satisfaction (score)

**Groups**	**Treatment Process**	**Service Duration**	**Operating Skill**	**Staff Attitude**	**Total Score**
**Control group**	3.3±0.7	3.1±0.6	2.4±0.4	3.0±0.5	11.5±2.3
**Observational group**	3.8±0.9	3.6±0.8	2.7±0.5	3.5±0.8	13.6±2.2
***t ***	0.123	0.185	0.202	0.326	4.865
***P ***	0.921	0.869	0.821	0.785	0.027

The emergency clinical nursing pathway mainly focuses on the integration of the emergency mode and chain rescue process.

The integration emergency mode is the integration of first aid, the emergency department, the emergency operating room, emergency intensive care unit, and emergency observation room. The main feature is underlined by the word “emergency” (3). The integration mode can be accurately, timely, and effectively respond to emergency diseases that can shorten the duration of first aid and hierarchical partition for the disease, and increase the successful treatment rate and satisfaction rate. The hierarchical partition management mode is a key for emergency nursing and determines the rescue process, first aid duration, and successful treatment rate. Therefore, it is a critical guideline for emergency surgery, observation, and registration order management (4).

It is important to make full use of limited medical resources and motivate health-care workers. It is important that health-care workers are responsible and have standardized understanding to make correct diagnoses and manage the rescue process to improve health care work quality (5). Different nursing levels and clinical nursing pathways have been adopted based on disease severity within hospital of pre-hospital, which can play an important role in optimizing the emergency process, improving humanized management, standardizing emergency medical treatment for specific diseases, and improving hospital services and innovation (6). Based on our study, the successful treatment rate in the observational group was increased. The nursing error rate was lower, and the emergency residence duration was shorter compared with the control group. In addition, the nursing satisfaction was improved.

Therefore, the clinical nursing pathway has significant application value in the classification treatment of patients with emergency dyspnea, and can significantly improve the treatment efficiency and nursing service level.

## References

[1] Banzett RB O’Donnell CR Guilfoyle TE (2015). Multidimensional Dyspnea Profile: an instrument for clinical and laboratory research. Eur Respir J, 45(6):1681–91. 25792641 10.1183/09031936.00038914 PMC4450151

[2] Lavin MA Krieger MM Meyer GA Spasser MA Cvitan T Reese CG Carlson JH Perry AG Mcnary P (2005). Development and evaluation of evidence-based nursing (EBN) filters and related databases. J Med Libr Assoc, 93(1): 104–115. 15685282 PMC545129

[3] Albrich WC Rüegger K Dusemund F Schuetz P Arici B Litke A Blum CA Bossart R Regez K Schild U (2013). Biomarker-enhanced triage in respiratory infections: a proof-of-concept feasibility trial. Eur Respir J, 42(4):1064–75. 23349444 10.1183/09031936.00113612 PMC3787815

[4] Dykes PC Wantland D Whittenburg L Lipsitz S Saba VK (2013). A Pilot Study to Explore the Feasibility of Using the Clinical Care Classification System for Developing a Reliable Costing Method for Nursing Services. AMIA Annu Symp Proc, 2013: 364–371. 24551343 PMC3900184

[5] Chang YC Ng CJ Wu CT Chen LC Chen JC Hsu KH (2013). Effectiveness of a five-level Paediatric Triage System: an analysis of resource utilisation in the emergency department in Taiwan. Emerg Med J, 30(9):735–9. 22983978 10.1136/emermed-2012-201362 PMC3756519

[6] Jordi K Grossmann F Gaddis GM Cignacco E Denhaerynck K Schwendimann R Nickel CH (2015). Nurses’ accuracy and self-perceived ability using the Emergency Severity Index triage tool: a cross-sectional study in four Swiss hospitals. Scand J Trauma Resusc Emerg Med, 23: 62. 26310569 10.1186/s13049-015-0142-y PMC4551516

